# Global trends and research frontiers on heat stress in poultry from 2000 to 2021: A bibliometric analysis

**DOI:** 10.3389/fphys.2023.1123582

**Published:** 2023-02-07

**Authors:** Victoria Anthony Uyanga, Taha H. Musa, Oyegunle Emmanuel Oke, Jingpeng Zhao, Xiaojuan Wang, Hongchao Jiao, Okanlawon M. Onagbesan, Hai Lin

**Affiliations:** ^1^ Shandong Provincial Key Laboratory of Animal Biotechnology and Disease Control and Prevention, Key Laboratory of Efficient Utilization of Non-Grain Feed Resources (Co-Construction by Ministry and Province), Ministry of Agriculture and Rural Affairs, College of Animal Science and Technology, Shandong Agricultural University, Tai’an, China; ^2^ Biomedical Research Institute, Darfur University College, Nyala, Sudan; ^3^ Department of Animal Physiology, Federal University of Agriculture, Abeokuta, Nigeria

**Keywords:** bibliometric, chickens, growth performance, heat stress, oxidative stress, VOSviewer

## Abstract

**Background:** Heat stress remains a major environmental factor affecting poultry production. With growing concerns surrounding climate change and its antecedent of global warming, research on heat stress in poultry has gradually gained increased attention. Therefore, this study aimed to examine the current status, identify the research frontiers, and highlight the research trends on heat stress in poultry research using bibliometric analysis.

**Methods:** The literature search was performed on the Web of Science Core Collection database for documents published from 2000 to 2021. The documents retrieved were analyzed for their publication counts, countries, institutions, keywords, sources, funding, and citation records using the bibliometric app on R software. Network analysis for co-authorship, co-occurrence, citation, co-citation, and bibliographic coupling was visualized using the VOSviewer software.

**Results:** A total of 468 publications were retrieved, and over the past two decades, there was a gradual increase in the annual number of publications (average growth rate: 4.56%). China had the highest contribution with respect to the number of publications, top contributing authors, collaborations, funding agencies, and institutions. Nanjing Agricultural University, China was the most prolific institution. Kazim Sahin from Firat University, Turkey contributed the highest number of publications and citations to heat stress in poultry research, and Poultry Science was the most productive and the most cited journal. The top 10 globally cited documents mainly focused on the effects of heat stress, alleviation of heat stress, and the association between heat stress and oxidative stress in poultry. All keywords were grouped into six clusters which included studies on “growth performance”, “intestinal morphology”, “heat stress”, “immune response”, “meat quality”, and “oxidative stress” as current research hotspots. In addition, topics such as; “antioxidants”, “microflora”, “intestinal barrier”, “rna-seq”, “animal welfare”, “gene expression”, “probiotics”, “feed restriction”, and “inflammatory pathways” were identified for future research attention.

**Conclusion:** This bibliometric study provides a detailed and comprehensive analysis of the global research trends on heat stress in poultry over the last two decades, and it is expected to serve as a useful reference for potential research that will help address the impacts of heat stress on poultry production globally.

## 1 Introduction

With the imminent challenge of climate change and its antecedent of global warming, the growing increase in ambient temperature affects all life forms including humans, plants, and animals. This raises a major concern about the extent to which the ongoing variability in climatic conditions would directly and/or indirectly affect the future of animal production (Oke et al., 2021). Heat stress is a significant environmental stressor, especially in the tropics and sub-tropical regions of the world. It occurs under prevailing high temperatures where the animal cannot dissipate its body temperature to the surrounding environment, causing a negative balance between the amount of heat generated and the body’s heat loss (Lara and Rostagno, 2013). Heat stress largely affects poultry production since modern-day birds are highly vulnerable to high temperatures and they have limited heat dissipation capacity (Uyanga et al., 2022a). The feather coverage of birds and their lack of sweat glands makes them prone to high ambient temperatures relative to other monogastric animals (Zaboli et al., 2019). Thus, the incidence of heat stress is associated with severe detrimental effects on the health, welfare, and productivity of poultry species, accruing to significant economic losses (Oke et al., 2021; Madkour et al., 2023). Importantly the effects of heat stress on poultry production are directly associated with food safety issues, thus warranting special attention in order to bridge the protein demand to supply gap.

Studies have shown that heat stress negatively affects growth performance, production indices, behavior, immunity, metabolism, welfare, and physiological responses of poultry (Azad et al., 2010; Soleimani et al., 2011; Farag and Alagawany, 2018). It is characterized by an increase in body temperature, higher respiratory rate, decreased feed intake, lowered body weight, poor feed efficiency, low egg production, decreased reproduction, impaired immunity, impaired metabolism, alterations in intestinal microflora, poor intestinal morphology, increased inflammatory response, increased production of reactive oxygen species, deteriorated meat quality, and in extreme cases, results to death of the animals (Wang et al., 2018; Liu et al., 2020; Alagawany et al., 2021; Nawaz et al., 2021; Uyanga et al., 2021; Madkour et al., 2022). The effects of heat stress have been studied on different poultry species, and the findings reported are multifaceted, and interwoven. To better understand the impacts of heat stress in poultry and the strategies for its alleviation, various studies have been conducted under varied experimental conditions. Several works have extensively reviewed the impacts of heat stress and the approaches for its mitigation in poultry. It can be surmised that these mitigating approaches center on environmental modification, management techniques, nutritional manipulation, genetic manipulation, and perinatal conditioning of the birds (Lin et al., 2006; Saeed et al., 2019; Wasti et al., 2020; Abdel-Moneim et al., 2021; Kumar et al., 2021; Uyanga et al., 2022b; Madkour et al., 2023). Therefore, it is evident that a reasonable number of scientific information has been generated on heat stress in poultry, however, to the best of our knowledge, there currently exists no bibliometric study that has comprehensively examined the available literature to stimulate further research in this area.

Bibliometric analysis has been described as a scientific methodology that utilizes computer-assisted review to examine all the publications on a specific topic or field in order to identify the core research, authors of the subject, and their relationships over a given period (Nicolaisen, 2010; Liu et al., 2022). The conduct of bibliometric analysis provides information on the topic of interest and further gives an overall understanding of the intellectual landscape. Attributes such as the author or citation information, titles, keywords, and abstract data have been developed for network analysis and sociometric analysis (Han et al., 2020; Musa et al., 2020). Bibliometric is increasingly important in managing the increasing number of academic publications which often involve empirical contributions producing voluminous, fragmented, and controversial research outcomes (Aria and Cuccurullo, 2017). Compared to other scientific review techniques, bibliometric provides highly objective and reliable analyses since it employs a “systematic, transparent, and reproducible review process that is based on the statistical measurement of science, scientists, or scientific activity” (Wallin, 2005). Therefore, with the increasing number of research reporting on heat stress in poultry production, it was deemed necessary to provide evidence-based insights *via* a bibliometric analysis of the research productivity by authors, countries, keywords, funding agencies, and collaboration networks. The findings of this study would help unveil the research progress on heat stress in poultry and its development in recent decades. This would also provide organized information for researchers, poultry industry experts and further advance research in this area.

Therefore, this bibliometric study was conducted to investigate, identify and visualize the network of publications that have shaped the intellectual discourse and research structure on heat stress in poultry from 2000 to 2021. The mapping of the thematically related publications and an examination of the contributions, co-operations, and recent trends as it relates to the heat stress in poultry was also carried out.

## 2 Materials and methods

### 2.1 Data sources

Relevant documents for the bibliometric study were retrieved from the Web of Science Core Collection (WoSCC). This database is considered appropriate to retrieve eligible literature on the subject matter in an appropriate reference format. WoSCC is the world’s foremost citation database covering articles from over 21,000 peer-reviewed journals, with about 1.9 billion cited references. It is a reliable database and is widely utilized for bibliometric studies in various disciplines (Chen et al., 2021; Cheng et al., 2022; Shan et al., 2022)

### 2.2 Search strategy

For this study, published peer-reviewed articles on heat stress and poultry were retrieved from the WoSCC database through a comprehensive search of online documents published from 2000 to 2021. The search was carried out by two investigators (VAU and THM) on a single day, 10 October 2022 to eliminate variations arising from database updates. To build a valid search query that will retrieve as many relevant documents as possible, specific keywords were used. Several “systematic reviews” and “bibliometric analyses” articles were reviewed to build a search query for heat stress and poultry production (Liu et al., 2020; Leishman et al., 2021; Siddiqui et al., 2022).

The search strategy was as follows:

#1: “poultry” (Title) OR “chicken” (Title) OR “guinea fowl” (Title) OR “turkey” (Title) OR “geese” (Title) OR “duck” (Title) OR “quail” (Title) OR “pigeon” (Title) OR “broilers” (Title) OR “laying hen” (Title) OR “fowl” (Title) OR “ostriches” (Title) OR “pheasants” (Title)
#2: “heat stress” (Title) OR “thermal stress” (Title) OR “high temperature” (Title) OR “hot temperature” (Title) OR “thermal condition” (Title) OR “high ambient temperature” (Title) OR “heat exposure” (Title) OR “heat conditioning” (Title) OR “high environmental temperature” (Title) OR “high environmental temperature” (Title). The two queries were combined to generate the final dataset: #1 and #2. Details of the search methodology are provided as Supplementary Material.

**FIGURE 1:**
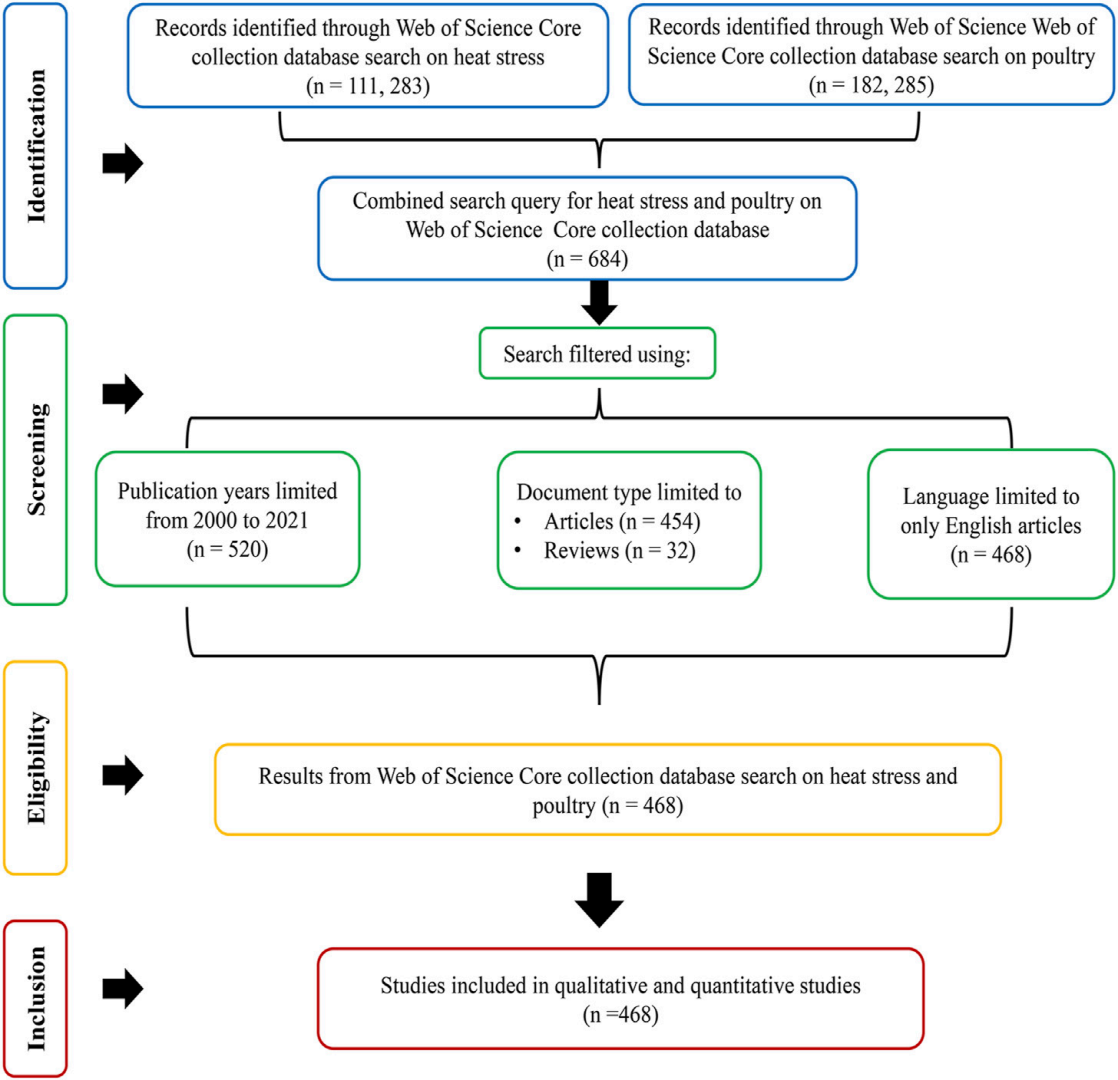
Schematic illustration of the search strategy conducted to retrieve published articles on heat stress in poultry from 2000 to 2021.

The quotation mark (“ ”) was used as the Boolean search modifier to retrieve exact phrases for search terms and to avoid the split up of search terms that contained more than one word/phrase into single-word components. The search query was built mainly on the “Title” field tag to ensure that the retrieved documents were directly related to heat stress and poultry. The time span was limited to the period of research interest, from 2000 to 2021. Publication type was limited to “articles” and “review, and document type was restricted to only English language. A schematic illustration of the search strategy is presented in Figure 1.

### 2.3 Data extraction

The retrieved documents were extracted using the “Export Records to File” button and the “Full Records and Cited References” option on WoSCC. The plain text, RIS, BibTeX, excel, and tab-delimited files were downloaded for further processing. The exported data included information on authors, publication years, document types, web of science categories, affiliations, journals, funding agencies, countries, research areas, citations, and keywords. The journal impact factor (IF) and category quartile were obtained from the 2021 Journal Citation Reports. The quality of publications was evaluated using the number of citations and the Hirsh-index (h-index).

### 2.4 Bibliometric analysis

Qualitative and quantitative analysis was performed using the Biblioshiny app on R studio (version 2022.07.1 + 554) (Aria and Cuccurullo, 2017). This platform was used to generate results for main publication information, annual production and citations, author’s impact, relevant affiliations, relevant sources, most cited countries, WordCloud analysis, thematic evolution, and factorial analysis. The bibliometric online analysis platform (https://bibliometric.com), was used to analyze and visualize the partnership analysis between countries.

The VOSviewer software (version 1.6.11) developed by Nees Jan van Eck and Ludo Waltman (van Eck and Waltman, 2010), was used for creating, visualizing, and exploring bibliometric maps based on network data. VOSviewer was used to analyze the co-authorship of authors, countries, and institutions; co-occurrence of all keywords; citation analysis of documents, sources, authors, institutions, and countries; bibliographic coupling of documents, sources, authors, and countries; and the co-citation analysis of cited references, cited sources and cited authors. The network data were generated as network visualization and overlay visualization and interpreted accordingly. The nodes on the maps were representative of the analyzed element (that is, author, country, institution, source, or document). The line between nodes represents the link between two elements, and the thickness of the lines represents the link strength of their connections. The size of an element was determined based on its total link strength (TLS), and different colors were used to differentiate elements into different clusters (van Eck and Waltman, 2010; Cheng et al., 2022).

### 2.5 Data analysis

R software (version 4.2.1), GraphPad Prism (version 8.0.2), and Microsoft Excel 2016 were used to perform descriptive statistics and plot charts. The Spearman correlation coefficients were conducted using GraphPad Prism. Data were considered statistically significant at *p* < 0.05.

## 3 Results

### 3.1 Annual growth of publication and citations

A total of 468 documents on heat stress and poultry research. These included 93.16% of articles and 6.84% of reviews from 144 sources. The average citation per document was 24.11, and the documents had an average age of 6.12 years. The studies involved a total of 1, 679 authors, with 13 single-authored documents and 5.2 co-authors per document. The results revealed an international collaboration rate of 24.36% among the authors as shown in Table 1.

**TABLE 1:** Main characteristics of the published articles on heat stress and poultry.

Description	Results
Timespan	2000:2021
Document sources (Journals, Books, etc.)	144
Number of documents	468
Annual growth rate (%)	4.56
Document average age	6.12
Average citations per document	24.11
References	13,844
Document contents	
Keywords plus	1,381
Author’s keywords	1,068
Authors	
Authors	1,679
Authors of single-authored documents	13
Author Productivity through Lotka’s Law (no. of authors)	
Document written by one author	1,333
Document written by two author	207
Document written by three author	60
Authors collaboration	
Single-authored documents	13
Co-authors per documents	5.2
International co-authorships (%)	24.36
Document types	
Article	436
Review	32

**FIGURE 2:**
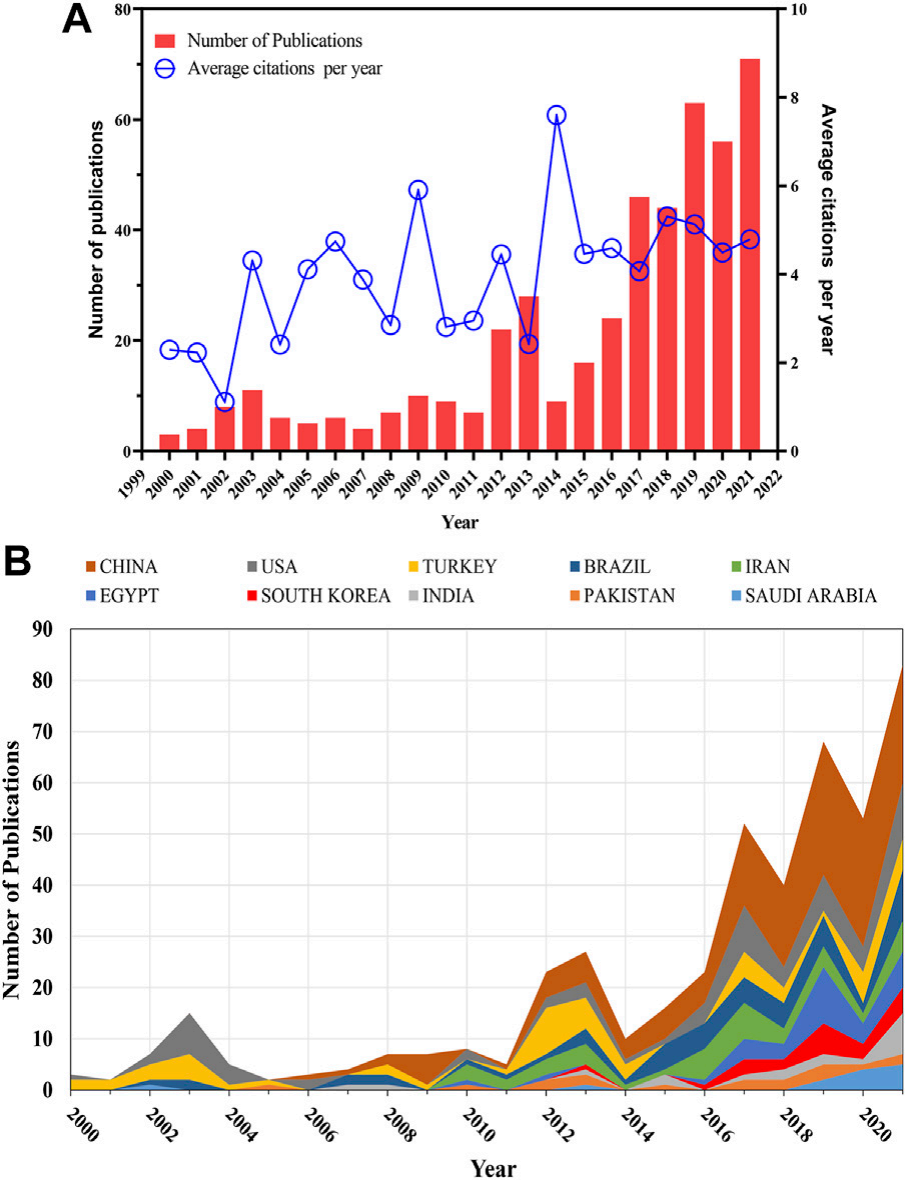
Trends in annual publications on heat stress in poultry. **(A)** Annual number of publications and average citation of articles. **(B)** Annual publication trends from top productive countries.

The annual publications and average citations from 2000 to 2021 are shown in Figure 2A. The annual production of publications on heat stress in poultry rose steadily from three documents as at 2000 to 79 documents in 2021, with an annual growth rate of 4.56%. Correspondingly, the average citations increased from 2.29 in 2000 to the highest point of 7.60 in 2014, and it was at 4.79 in 2021. A significant correlation coefficient was established between the number of publications and the average number of citations for the past 22 years (r = 0.5438, *p* > 0.0089). Figure 2B shows the United States dominated the number of publications until 2005. However, China made significant developments from 2006 and has increasingly dominated the number of publications in this field.

### 3.2 Contribution of funding agencies and institutions

**FIGURE 3:**
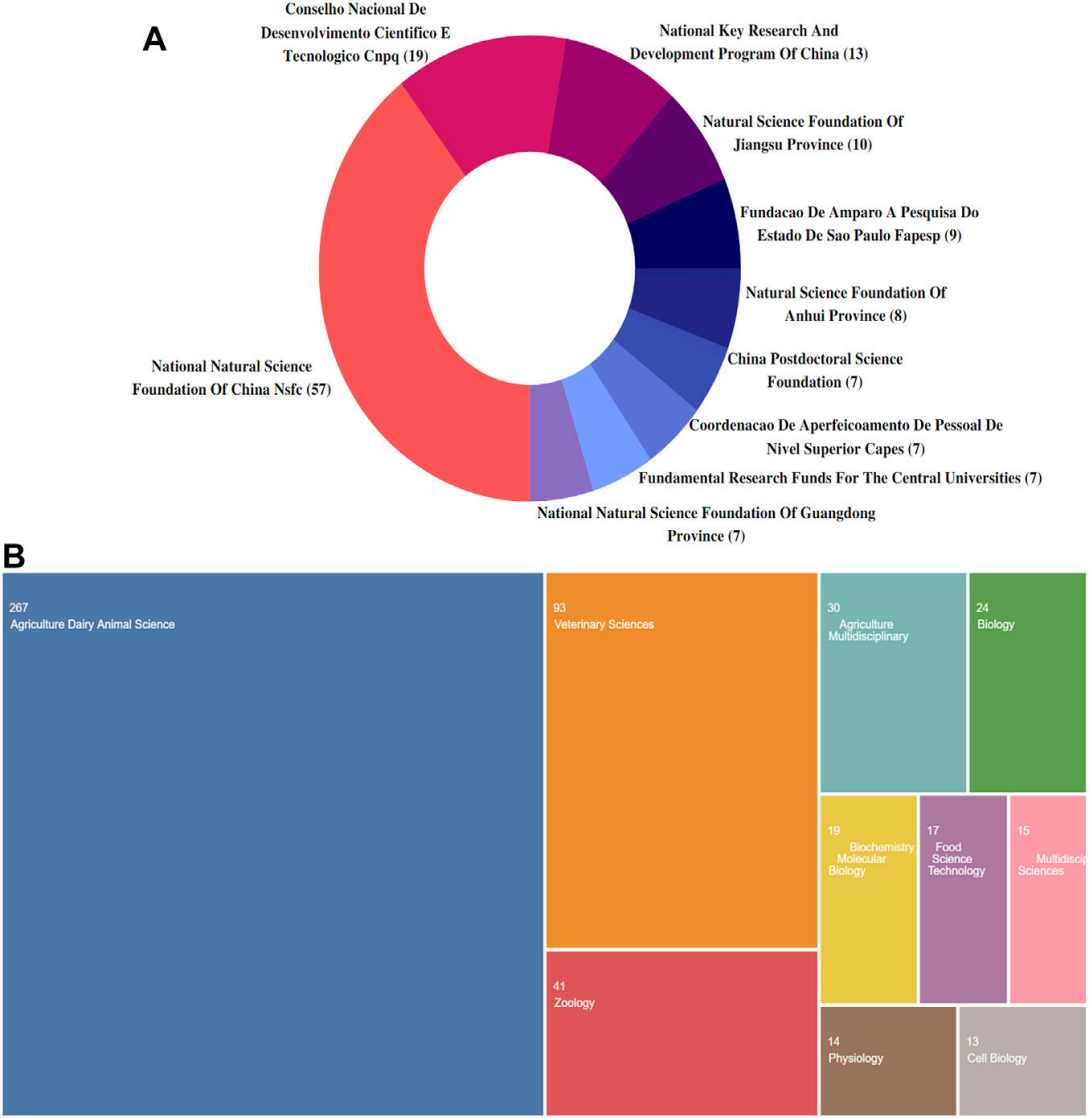
Overview of global research on heat stress in poultry. **(A)** Contribution of various funding bodies. **(B)** Research areas according to subject categories.

A summary of the top 10 most active funding agencies is shown in Figure 3A. Five of these were from China and the top three funding agencies that supported research on heat stress in poultry were the National Natural Science Foundation of China (n = 57, 12.18%), Conselho Nacional De Desenvolvimento Cientifico E Tecnologico (n = 19, 4.06%), and National Key Research and Development Program of China (n = 13, 2.78%). Figure 3B shows that among the WoSCC subject categories, the top three areas with the highest contribution to heat stress in poultry were Agriculture, Dairy, Animal Science (n = 267), Veterinary Science (n = 93), and Zoology (n = 41).

**FIGURE 4:**
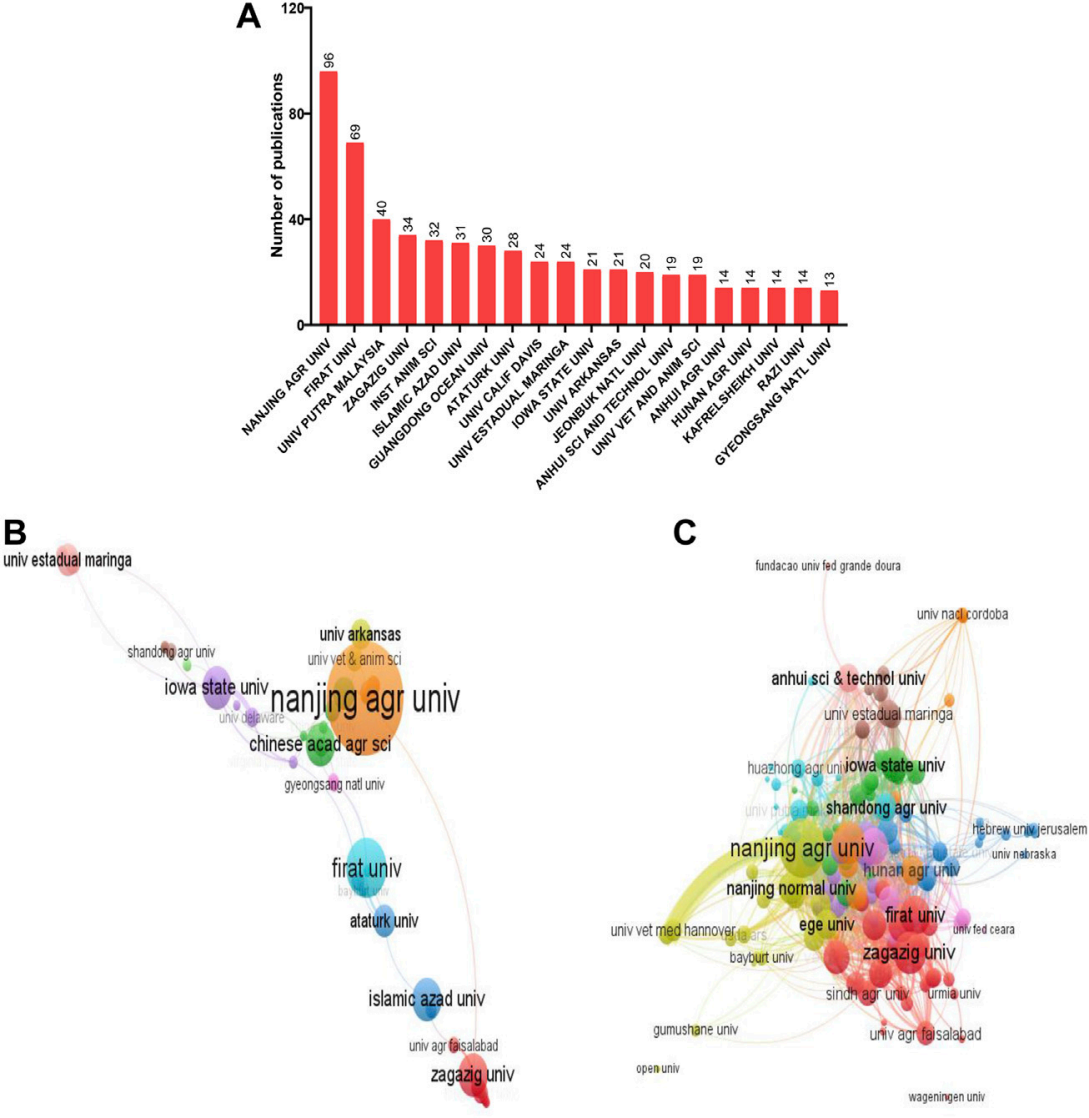
Analysis on the institutional contributions to research on heat stress in poultry. **(A)** Most relevant affiliations. **(B)** Network visualization for co-authorship analysis of contributing institutions. **(C)** Network visualization for the citation analysis of contributing institutions. *The larger the node, the greater the institutional contribution, and the thicker the line, the greater the link strength between the connected institutions.*

As shown in Figure 4A, the most relevant institutions that published on heat stress in poultry were majorly from China (25%) and the United States (15%). The top institutions with the highest number of publications were Nanjing Agricultural University (China, n = 96), Firat University (Turkey; n = 69), and Universiti Putra Malaysia (Malaysia, n = 40). Co-authorship analysis of the author’s institutions (minimum of three documents) showed that 58 institutions formed the largest set of connections (Figure 4B). A total of 11 institutional clusters were formed with five links and 151 TLS. In the network visualization map, Nanjing Agricultural University had the largest node with the greatest total link strength (TLS = 26). Other top collaborating institutions were Iowa State University (TLS = 19), University Estadual Maringa (TLS = 14), Firat University (TLS = 13), and Zagazig University (TLS = 12).

For the citation analysis, a total of 139 institutions (minimum number of documents of two) met the threshold and formed the largest connection (Figure 4C). The density between the institutions showed that they were closely related in terms of their citation links. Key institutions were Nanjing Agricultural University (TLS = 503), Zagazig University (TLS = 242), Firat University (TLS = 200), Iowa State University (TLS = 150), and Nanjing Normal University (TLS = 130). Importantly, it was observed that Nanjing Agricultural University had the greatest citation linkages with the Chinese Academy of Agricultural Sciences, Anhui University of Science and Technology, and University of Veterinary Medicine Hanover.

### 3.3 Contribution of countries

**FIGURE 5:**
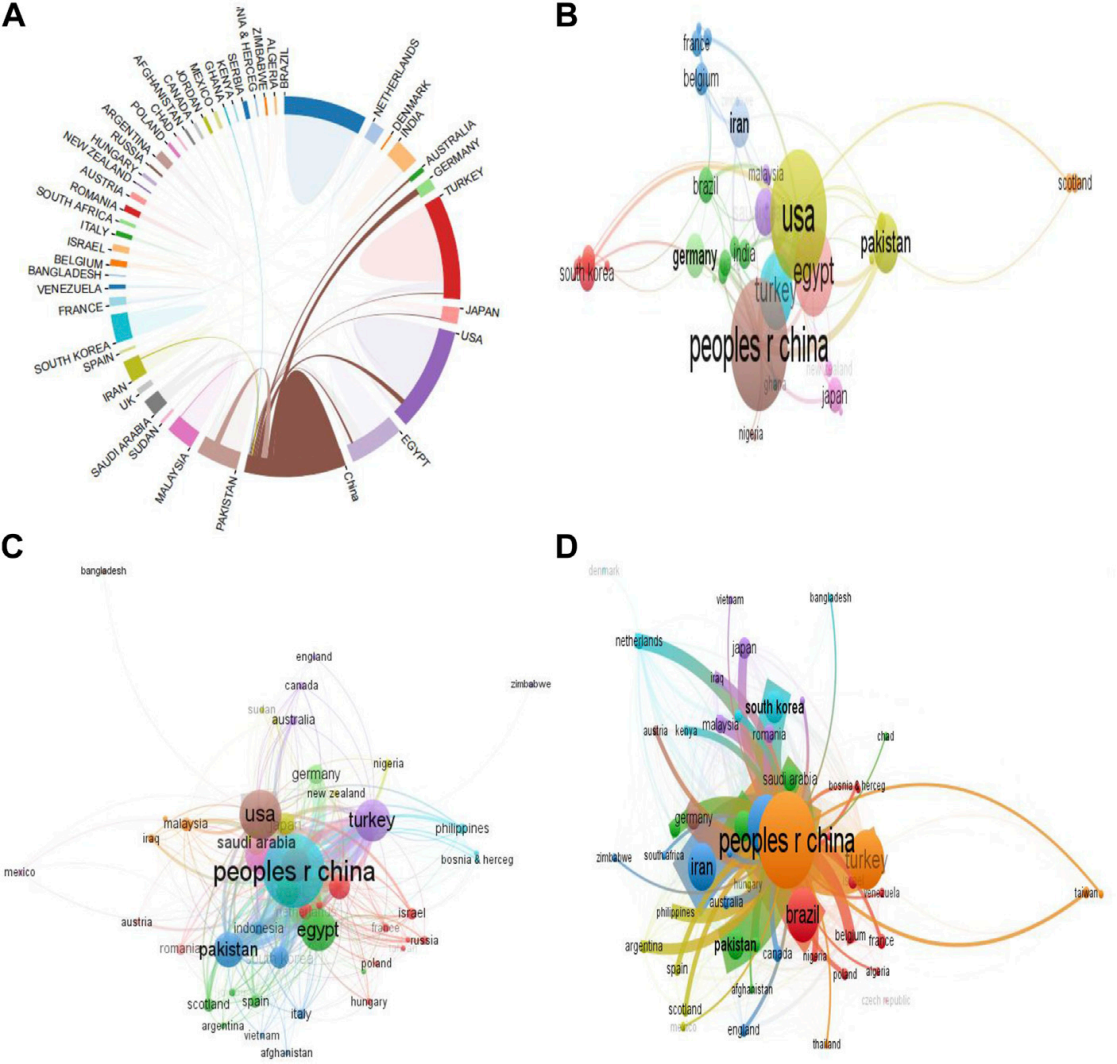
Countries contributions to research on heat stress in poultry. **(A)** Interrelationship between contributing countries. **(B)** Co-authorship mapping of countries. **(C)** Citation analysis of countries. **(D)** Bibliographic coupling of countries. *The larger the node, the greater the country’s contribution, and the thicker the line, the greater the link strength between two connected countries.*

From Table 2, analysis for the most relevant country based on the corresponding authors revealed that China had the highest number of publications, followed by Turkey, Brazil, United States, and Iran. These accrued from both single- and multi-country publications. Interestingly, the multiple-country publication (MCP) analysis showed that France (MCP_Ratio:0.750) and the Netherlands (MCP_Ratio: 0.750) had the highest collaboration ratio (data not shown). Among the top 20 most cited countries, China had the highest total citations, followed by Turkey, United States, Iran, and Japan. Correspondingly, the collaboration network among contributing countries showed that China had close collaboration with Unites States, Germany, Australia, Egypt, Japan, and Turkey (Figure 5A).

**TABLE 2:** Top 10 productive countries contributing to research on heat stress in Poultry.

	Corresponding author’s country							Most cited countries		
Rank	Country		Articles	SCP	MCP	Freq	MCP ratio	Country	TC	AAC
1	China		137	113	24	0.293	0.175	China	3,912	28.55
2	Turkey		56	42	14	0.120	0.250	Turkey	1,671	29.84
3	Brazil		49	45	4	0.105	0.082	United States	1,288	29.95
4	United States		43	30	13	0.092	0.302	Iran	706	20.17
5	Iran		35	32	3	0.075	0.086	Japan	445	49.44
6	Egypt		23	14	9	0.049	0.391	Brazil	435	8.88
7	Korea		16	12	4	0.034	0.250	Egypt	387	16.83
8	India		15	14	1	0.032	0.067	Pakistan	384	48.00
9	Japan		9	4	5	0.019	0.556	Belgium	321	80.25
10	Pakistan		8	5	3	0.017	0.375	Israel	180	36.00

AAC, average article citations; MCP, multiple country publication; SCP, single country publication; TC, total citations.

Co-authorship analysis of contributing countries showed that 46 countries constituted the largest connection (Figure 5B). They were grouped into 12 clusters, having 10 links and 178 TLS. Prominent countries with the largest connections were the People’s Republic of China (TLS = 45), United States (TLS = 44), Egypt (TLS = 29), Pakistan (TLS = 18), and Saudi Arabia (TLS = 14). The network analysis revealed that China had great co-authorship linkages with the United States, Pakistan, Germany, and Egypt. Correspondingly, the citation analysis showed that 49 countries were connected, with a total TLS of 265, 42 links, and were grouped into 11 clusters. The five most prominent countries were the People’s Republic of China (TLS = 1,055), United States (TLS = 538), Turkey (TLS = 413), Egypt (TLS = 416), and Pakistan (TLS = 314) (Figure 5C). Evidently, China had greater citation linkages with United States, Pakistan, Iran, Egypt, and Turkey.

In addition, the bibliographic coupling analysis of contributing countries (Figure 5D) showed that all 54 countries were connected and were categorized into 8 clusters having 109 links and 108, 141 TLS. Among the countries, People’s Republic of China (TLS = 40 822), United States (TLS = 20,421), Egypt (TLS = 20,213), Iran (TLS = 14,097), and Turkey (TLS = 13,270) had the highest TLS. Importantly, the People’s Republic of China was able to establish strong connections with countries such as United States, Brazil, Pakistan, Turkey, Iran, and South Korea.

### 3.4 Document sources

A total of 144 sources of documents were identified in this study. The top 20 journals with publications on heat stress in poultry are shown in Table 3. Based on the number of articles produced the most prolific journal was Poultry Science. This was followed by British Poultry Science, Journal of Thermal Biology, Animals, and Brazilian Journal of Poultry Science. Correspondingly, the total citations increased with these journals (Poultry Science and British Poultry Science), except that Worlds Poultry Science Journal had higher citations than Journal of Thermal Biology, which was followed by Biological Trace Element Research. Among these top 20 journals, the Journal of the Science of Food and Agriculture had the highest impact factor, and 60% of these journals were partitioned into JCR Q1.

**TABLE 3:** Top 20 most productive journals contributing to research on heat stress in poultry.

Rank	Journal	Number of articles	Total citation	h_index	IF	Rank	Journal
1	Poultry Science	66	3,067	28	4.014	4.192	Q1
2	British Poultry Science	23	768	13	1.892	2.429	Q2
3	Journal of Thermal Biology	17	524	12	3.189	3.342	Q1
4	Animals	16	302	9	3.231	3.312	Q1
5	Brazilian Journal of Poultry Science	15	170	9	1.019	1.492	Q3
6	Worlds Poultry Science Journal	12	593	10	3.452	3.762	Q1
7	Biological Trace Element Research	11	445	9	4.081	3.755	Q2
8	Plos One	10	333	8	3.752	4.069	Q1
9	Cell Stress and Chaperones	9	160	7	3.827	3.940	Q3
10	Asian-Australasian Journal of Animal Sciences	9	89	6	2.694	2.816	Q1
11	Animal Science Journal	8	180	5	1.974	2.027	Q2
12	Journal of Applied Poultry Research	8	104	5	2.162	2.000	Q2
13	Italian Journal of Animal Science	8	37	4	2.552	2.785	Q1
14	Tropical Animal Health and Production	8	109	4	1.893	1.930	Q2
15	Animal Feed Science and Technology	6	267	6	3.313	3.914	Q1
16	Journal of Animal Physiology and Animal Nutrition	5	209	5	2.718	2.747	Q1
17	Journal of the Science of Food and Agriculture	5	158	5	4.125	4.096	Q1
18	International Journal of Biometeorology	5	235	4	3.738	3.951	Q2
19	Animal	4	40	4	3.730	3.908	Q1
20	Journal of Animal Science	4	118	4	3.338	3.243	Q1

**FIGURE 6:**
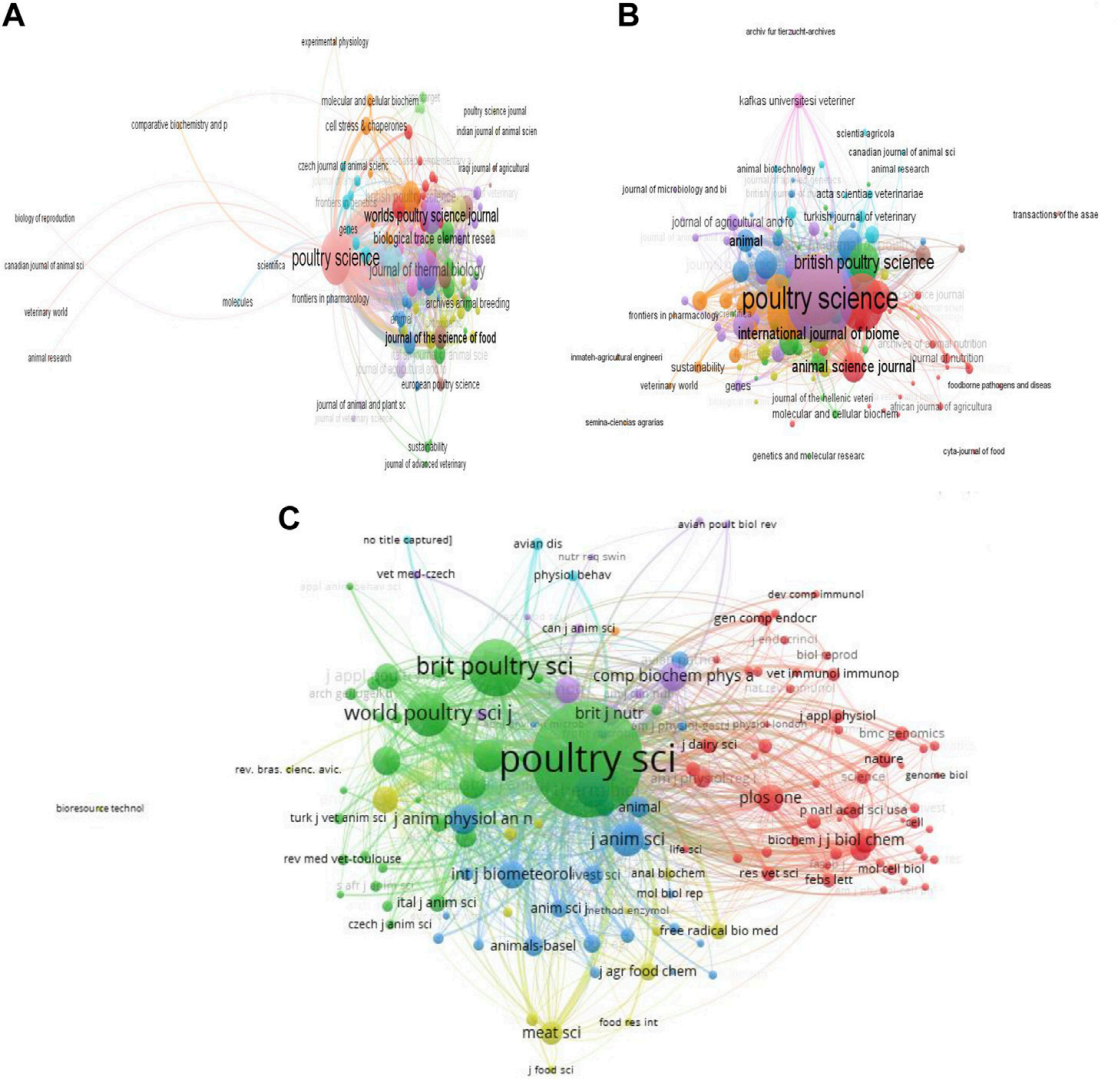
Journals contributing to research on heat stress in poultry. **(A)** Citation analysis of sources. **(B)** Bibliographic coupling of sources. **(C)** Co-citation analysis of cited sources. *The larger the node, the greater the journal’s contribution, and the thicker the line, the greater the link strength between two journals.*

A total of 111 sources formed the largest set of connections for citation analysis (Figure 6A). These sources had 681 links and 1,572 TLS. They were grouped into 17 clusters and the document sources with the strongest TLS were Poultry Science (TLS = 574), Journal of Thermal Biology (TLS = 243), World’s Poultry Science Journal (TLS = 207), Animals (TLS = 150) and British Poultry Science (TLS = 146). Interestingly, Poultry Science journal formed the greatest citation network with co-journals including Journal of Thermal Biology, British Poultry Science, World’s Poultry Science Journal, and Animals. The bibliographic coupling of sources showed that 128 sources formed the largest bibliographic connection with 4,262 links, TLS of 55,653 and they were grouped into 9 clusters (Figure 6B). Poultry Science had the largest network (TLS = 13,909), followed by Journal of Thermal Biology (TLS = 9,995), World’s Poultry Science Journal (TLS = 7,046), Animals (TLS = 6,092), and British Poultry Science (TLS = 3,719). Similar to the citation analysis, it was revealed that Poultry Science established strong bibliographic coupling with co-journals including Journal of Thermal Biology, World’s Poultry Science Journal, Animals, and British Poultry Science.

In total, 142 sources of documents (minimum number of citations per source = 20) were analyzed for the co-citation network of their cited sources (Figure 6C). Similar to the citation and bibliographic coupling networks, Poultry Science journal had the largest network of co-cited sources (TLS: 115,035). Other top co-cited sources were British Poultry Science (TLS = 31,960), World’s Poultry Science Journal (TLS = 20,901), Journal of Thermal Biology (TLS = 13,199), and Journal of Animal Science (TLS = 12,769). Importantly, Poultry Science had a great co-citation network with other journals especially, British Poultry Science, World’s Poultry Science Journal, International Journal of Poultry Science, and Journal of Animal Science and Biotechnology.

### 3.5 Authorship analysis

A total of 1,679 authors contributed to the 468 documents on heat stress in poultry from 2000 to 2021. Table 4 shows that the top five authors with the highest global contributions were Kazim Sahin (Firat University, Turkey), followed by Nurhan Sahin (Firat University, Turkey), Feng Gao (Nanjing Agricultural University, China), Li Zhang (Guangdong Ocean University, China) and Endong Bao (Nanjing Agricultural University, China). Interestingly, researchers from Nanjing Agricultural University, China constituted about 45% of the top 20 contributing authors, while China alone had 75% of the top 20 authors, followed by Turkey at 20%.

**TABLE 4:** Most productive authors contributing to global researches on heat stress in poultry.

Rank	Author	Institution	h_index	TC	NP
1	Kazim Sahin	Firat University, Turkey	14	736	17
2	Nurhan Sahin	Firat University, Turkey	11	482	13
3	Feng Gao	Nanjing Agricultural University, China	10	383	10
4	Li Zhang	Guangdong Ocean University, China	10	343	12
5	Endong Bao	Nanjing Agricultural University, China	9	262	14
6	Jiaolong Li	Nanjing Agricultural University, China	9	301	10
7	Shu Tang	Nanjing Agricultural University, China	8	150	13
8	Jiao Xu	Nanjing Agricultural University, China	8	147	11
9	Yun Jiang	Nanjing Normal University, China	7	268	7
10	Zhuang Lu	Nanjing Agricultural University, China	7	260	7
11	Bingbing Ma	Nanjing Agricultural University, China	7	268	7
12	Bin Yin	Shandong Academy of Agricultural Science, China	7	86	7
13	M. Zhang	Jinling Institute of Technology, China	7	161	13
14	Guanghong Zhou	Nanjing Agricultural University, China	7	254	7
15	Mohamed E. Abd El-Hack	Zagazig University, Egypt	6	208	6
16	Recep Gümüş	Cumhuriyet University, Turkey	6	102	6
17	Jianhua He	Hunan Agricultural University, China	6	210	8
18	Shaojun He	Anhui Science and Technology University, China	6	185	10
19	Xiaofang He	Nanjing Agricultural University, China	6	245	6
20	Halit İmik	Ataturk University, Turkey	6	102	6

NP, number of publications, TC: total citation.

**FIGURE 7:**
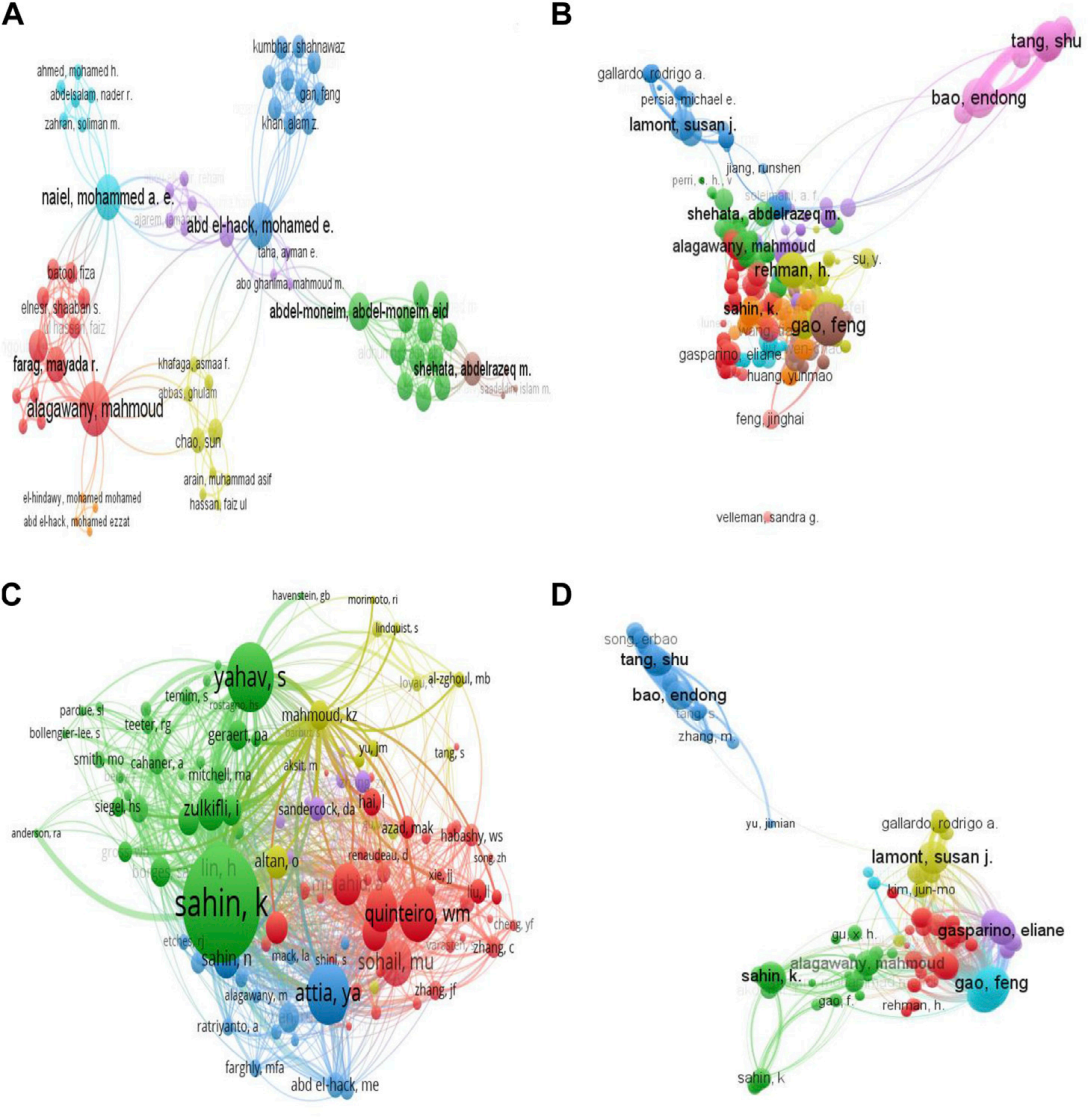
Analysis of the authorship network for global research on heat stress in poultry. **(A)** Co-authorship mapping of authors. **(B)** Citation analysis of authors. **(C)** Co-citation analysis of authors. **(D)** Bibliographic coupling analysis of authors. *The larger the node, the greater the author’s contribution, and the thicker the line, the greater the link strength between two authors.*

The co-authorship analysis showed that several of the authors were not connected, whereas the largest network had 69 authors, 304 links, and 309 TLS. Figure 7A shows that the five authors with significant co-authorship connections were Alagawany, Mahmoud (TLS = 26), Naiel Mohammed A.E (TLS = 21), Abd El-Hack, Mohamed E. (TLS = 21), Abdel-Moneim, Abdel-Moneim Eid (TLS = 17), and Shehata, Abdelrazeq M (TLS = 15). The co-authorship network revealed that of the few connected researchers, there was a close association between the different clusters. This suggests that the active authors on heat stress in poultry had established a centralized and concentrated network, but still lacked extensive collaboration with other scholars. Citation analysis of authors with a minimum of two documents showed that 308 authors formed the largest connection, with a total TLS of 10,244; 5,504 links, and they were categorized into 10 clusters (Figure 7B). Five authors with the strongest TLS were Gao, Feng (TLS = 366), Bao, Endong (TLS = 331), Tang, Shu (TLS = 282), Lamont, Susan J (TLS = 193), and Alagawany Mahmoud (TLS = 160). The co-citation network of cited authors revealed that a total of 115 authors were connected (minimum number of citations per author: 20) with 5,440 links; 42; 054 TLS, and they formed 5 clusters (Figure 7C). Top co-cited authors in the network were Sahin, K (TLS = 5,081), Yahav, S. (TLS = 2,849), Attia, Y.A (TLS = 2,551), Quinteiro, W.M (TLS = 2,183), and Lin, H (TLS = 5,081). Results from both the citation and bibliographic coupling analysis showed that Bao, Endong, and Tang, Shu who are major contributors affiliated with Nanjing Agricultural University, China were isolated in a distinct cluster.

Network visualization map showing the bibliographic coupling of authors also revealed that a total of 125 authors were connected (minimum number of documents: 3), with 6,721 links, total TLS of 152,247, and that they were grouped into 6 clusters (Figure 7D). Gao, Feng (TLS = 7,084), Lamont, Susan (TLS = 6,529), Bao, Endong (TLS = 5,435), Gasparino, Eliane (TLS = 5,097), and Sahin K (TLS = 5,054) were among the authors with the greatest TLS.

### 3.6 Documents and co-cited documents

The top 10 most globally cited documents on heat stress in poultry are presented in Table 5. “Effect of heat stress on oxidative stress, lipid peroxidation and some stress parameters in broiler” by Altan O. et al. (2003) was the most cited document. Two review articles were included in the top 10 globally cited documents. “Strategies for preventing heat stress in poultry” by Lin H et al. (2006) and “Association between heat stress and oxidative stress in poultry; mitochondrial dysfunction and dietary interventions with phytochemicals” by Akbarian A. et al. (2016).

**TABLE 5:** Top 10 most globally cited documents on heat stress in poultry.

Rank	Title	First author	Type	Source	PY	TC	TCY
1	Effect of heat stress on oxidative stress, lipid peroxidation and some stress parameters in broiler	Altan O.	Article	British Poultry Science	2003	294	14.7
2	Strategies for preventing heat stress in poultry	Lin H.	Review	World’s Poultry Science Journal	2006	250	14.71
3	Effects of different levels of zinc on the performance and immunocompetence of broilers under heat stress	Bartlett JR.	Article	Poultry Science	2003	246	12.3
4	Association between heat stress and oxidative stress in poultry; mitochondrial dysfunction and dietary interventions with phytochemicals	Akbarian, A.	Review	Journal of Animal Science and Biotechnology	2016	219	31.29
5	Effect of a probiotic mixture on intestinal microflora, morphology, and barrier integrity of broilers subjected to heat stress	Song J.	Article	Poultry Science	2014	215	23.89
6	Effect of supplementation of prebiotic mannan-oligosaccharides and probiotic mixture on growth performance of broilers subjected to chronic heat stress	Sohail MU	Article	Poultry Science	2012	215	19.55
7	Superoxide Radical Production in Chicken Skeletal Muscle Induced by Acute Heat Stress	Mujahid A.	Article	Poultry Science	2005	206	11.44
8	Effect of Chronic Heat Exposure on Fat Deposition and Meat Quality in Two Genetic Types of Chicken	Lu Q.	Article	Poultry Science	2007	193	12.06
9	Effects of different levels of vitamin E on growth performance and immune responses of broilers under heat stress	Niu ZY.	Article	Poultry Science	2009	180	12.86
10	Alleviation of cyclic heat stress in broilers by dietary supplementation of mannan-oligosaccharide and Lactobacillus-based probiotic: Dynamics of cortisol, thyroid hormones, cholesterol, C-reactive protein, and humoral immunity	Sohail MU.	Article	Poultry Science	2010	159	12.23

PY, published year; TC, total citation; TCY, total citation per year.

**FIGURE 8:**
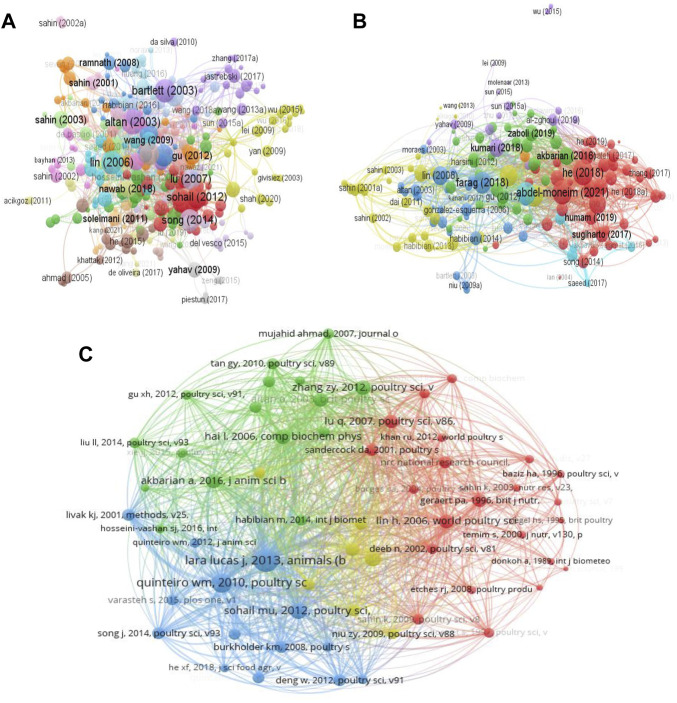
Network visualization of documents on heat stress in poultry. **(A)** Citation analysis of documents. **(B)** Bibliographic coupling analysis of documents. **(C)** Co-citation network of documents.

Following citation analysis (Figure 8A), the network visualization map showed that a total of 304 documents (minimum number of citations per document = 5) formed the largest set of connected items, which were grouped into 19 clusters. From the citation analysis, the following documents: Altan et al. (2003) (citations = 294, links = 39), Lin et al. (2006) (citations = 250, links = 35), Bartlett (2003) (citations = 246, links = 35), Akbarian et al. (2016) (citations = 219, links = 43), and Sohail et al. (2010) (citations = 215, links = 37) had the highest citation and linkages. Bibliographic coupling of documents revealed that 156 documents were connected (minimum number of citations per documents: 20) with a total TLS of 10,770, 406 links and they were distributed into 7 clusters (Figure 8B). The following; Wang et al. (2021) (TLS = 554), Abdel-Moneim et al. (2021) (TLS = 504), Farag and Alagawany (2018) (TLS = 430), Lin et al. (2006) (TLS = 338), and Kumar et al. (2021) (TLS = 325) were among the top documents with high TLS.

Analysis of the co-citation network of cited references (minimum number of citations per cited reference = 20) showed that 59 cited references formed a network of 1,548 links, 6,334 TLS and were grouped into 4 clusters (Figure 8C). Top five cited references included Lara and Rostagno (2013) (TLS = 1,377) which was published in Animals (Basel), followed by Quinteiro-Filho et al. (2010) (TLS = 994), Mashaly MM (2004) (TLS = 737), Sohail MU (2012) (TLS = 700), and Zhang ZY (2012) (TLS = 629). The last four cited references were all published in Poultry Science. Moreover, the strongest co-citation connection was found between the cited references, Lara and Rostagno (2013); and Quinteiro-Filho et al. (2010) (link strength = 37)

### 3.7 Keyword analysis

**FIGURE 9:**
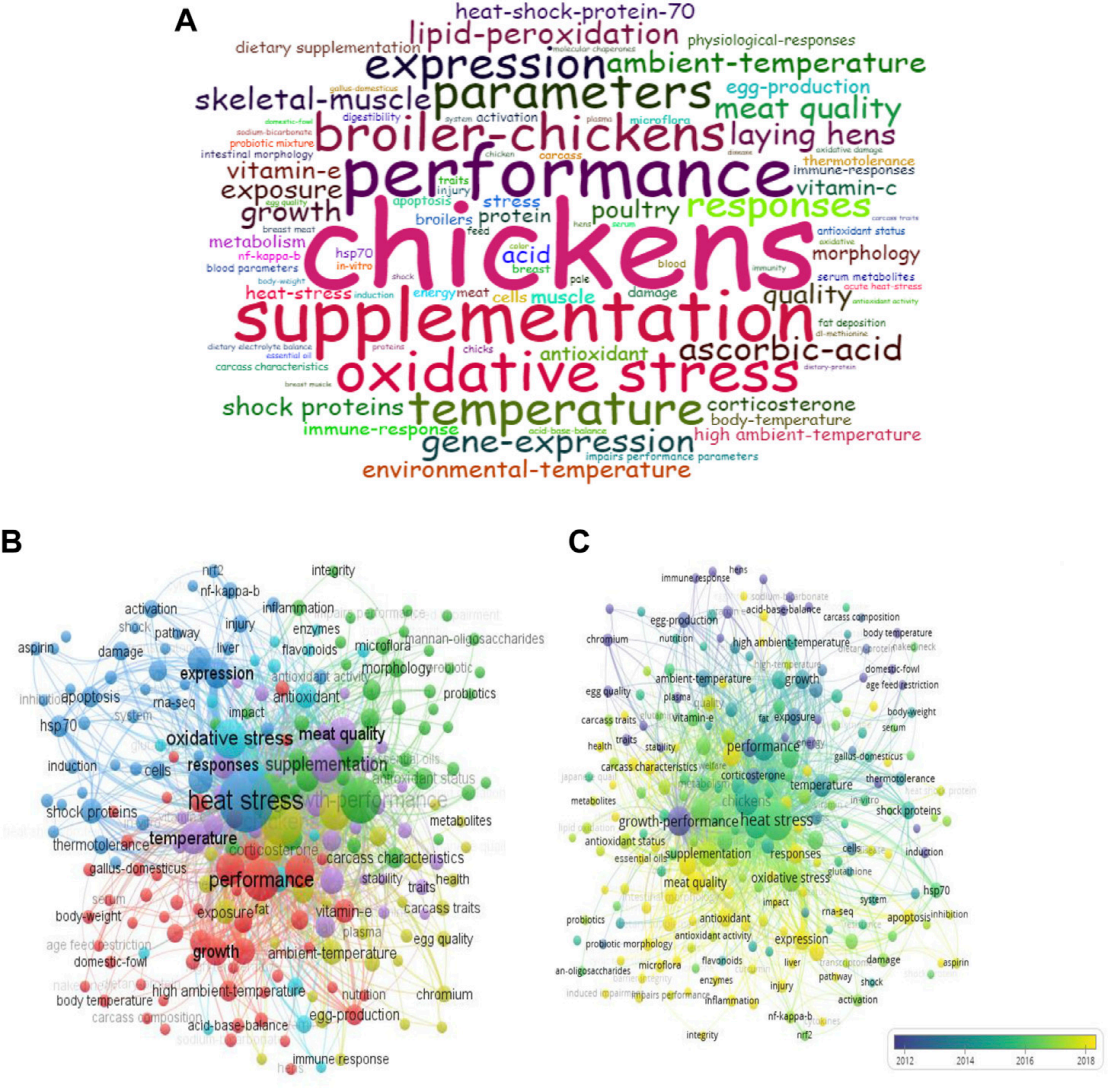
Analysis for Keywords associated with global research on heat stress in poultry. **(A)** Word cloud analysis of Keyword Plus. **(B)** Network visualization for all keywords co-occurrence. **(C)** Overlay visualization for the co-occurrence analysis of all keywords.

WordCloud analysis showed that “chickens” (127), “growth performance” (125), “supplementation” (75) “performance” (74), and “oxidative stress” (60) were the top five Keyword Plus with the highest frequency (Figure 9A). Co-occurrence analysis (minimum occurrence of each keyword = 5) categorized all the keywords into six clusters (Figure 9B). The top six keywords in terms of co-occurrence were “heat stress” (TLS = 1,884), “growth performance” (TLS = 1,027), “chickens” (TLS = 988), “broiler” (TLS = 961), “performance” (TLS = 781), and “oxidative stress” (TLS = 684) (Figures 9B, C). The overlay visualization showed that the keyword “heat stress” co-occurred with several current topics including “meat quality”, “antioxidants”, “microflora”, “intestinal barrier”, “expression”, and “rna-seq” (Figure 9C).

**FIGURE 10:**
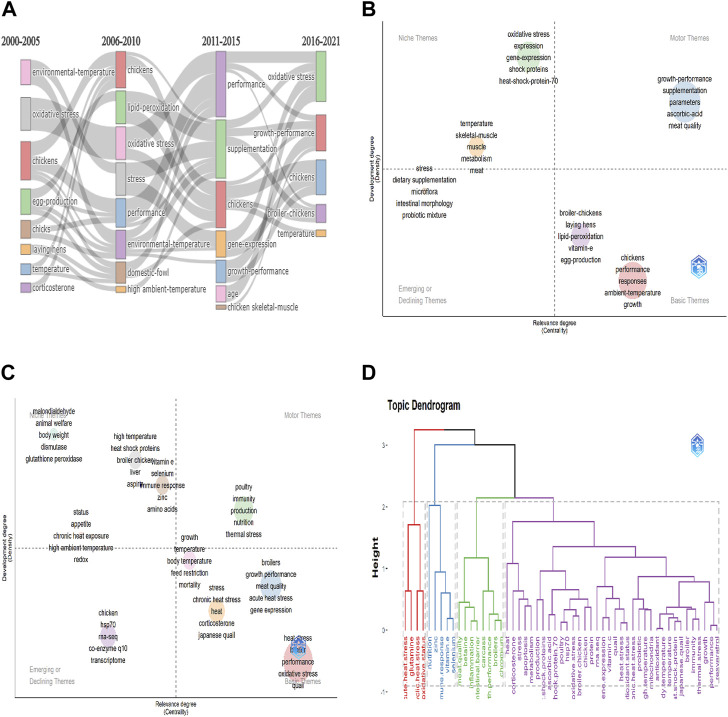
Keywords analysis for global research terms on heat stress in poultry. **(A)** Thematic evolution of Keyword Plus from 2000 to 2021. **(B)** Thematic map analysis of Keyword Plus **(C)** Thematic map analysis of authors keywords. **(D)** Topic dendrogram using multiple correspondence analysis of authors keywords to show the hierarchical relationship between terms.

To understand the research trend on heat stress in poultry over time, a thematic evolution analysis was conducted by grouping the Keyword Plus into four periods (2000–2005, 2006–2010, 2011–2015, and 2016–2021). Supplementary Table S1 gives detailed information on Keyword plus classification and the properties of the clusters/time series. As shown in Figure 10A, from 2000 to 2005 had key terms such as environmental temperature, oxidative stress, and chickens; 2006 to 2010 had terms such as lipid-peroxidation, oxidative stress; environmental temperature; 2011 to 2015 had high frequency for performance, supplementation, and chickens, and the period between 2016 and 2021 included terms such as oxidative stress, growth performance, and temperature.

Thematic map analysis was carried out for the Keyword plus and Author keywords (Figures 10B, C). For the Keywords plus, the motor theme covered terms such as growth performance, ascorbic acid, and meat quality. The niche themes were terms related to oxidative stress, gene expression, and muscle metabolism. The emerging or declining themes covered terms such as stress, dietary supplementation, microflora, intestinal morphology, and probiotic mixture, while the basic themes included terms related to broiler chickens, lipid peroxidation, and ambient temperature (Figure 10B). Thematic mapping of the Authors keywords showed that the motor theme included immunity, nutrition, and thermal stress. The niche theme had terms associated with oxidative stress, heat shock proteins, and chronic heat exposure. The emerging or declining themes featured terms such as chicken, hsp 70, rna-seq, co-enzyme 10, and transcriptome, while the basic theme had growth performance, terms related to heat stress, and gene expression (Figure 10C). Figure 10D shows the conceptual framework of Author keywords (n = 50) using the multiple correspondence analysis. The results showed that the keywords were categorized into four clusters of 4, 5, 8, and 33 elements, respectively. Key terms including “acute heat stress, “cyclic heat stress” and “oxidative status” were covered in the first cluster, whereas, the last cluster which had the largest number of elements (n = 33) covered terms closely related to heat stress, growth performance, gene expression, production, and metabolism.

## 4 Discussion

Over the last decades, with the increasing awareness and global actions towards climate change and global warming, research on heat stress in farm animals especially poultry has gained attention, as evidenced by the annual increment in the number of publications. The number of publications and citation counts achieved each year is an indicator of the research interest and progress made by researchers in that specific area (Musa et al., 2020; Xu et al., 2022). Several researchers, countries, institutions, and industry partners have collaborated on studies to address “heat stress in poultry”, thus necessitating this bibliometric analysis to understand the milestones achieved, research gaps uncovered, and the trends for future research. Over the past two decades, the annual publications on heat stress in poultry showed a significant increase, and so far, the highest number of articles was achieved in 2021. The annual number of publications increased relatively slowly from 2000 until 2011, with fewer than 15 articles per year. The increase observed from 2012 could possibly occur due to increasing awareness of the impacts of heat stress on poultry (Lin et al., 2006; Yahav, 2009) and its association with oxidative stress (Altan et al., 2003; Mujahid et al., 2005), as seen from the records of top globally cited documents. More so, the dietary manipulation of heat stress in poultry using prebiotics and probiotics (Lan et al., 2004; Sohail et al., 2010), polyphenols (Pena et al., 2008; Aengwanich and Suttajit, 2010), amino acids (Zarate et al., 2003; Dai et al., 2011), as well as, minerals and vitamins (Sahin et al., 2002; Niu et al., 2009; Mohiti-Asli et al., 2010; Vakili et al., 2010; Moeini et al., 2011) etc., were gradually gaining research attention. The last 5 years have witnessed production exceeding 40 documents annually, accounting for 61.9% of the total documents. The increase in the number of publications was positively associated with the rise in the average annual citation in a monotonic relationship. Also, it is expected that with an increase in the number of citable years, the average citation will continue to increase. Therefore, consistent efforts to foster research and collaboration are necessary to drive annual publications, growth rate, and article citations on heat stress and poultry.

Importantly, China ranked foremost in its contribution to research on heat stress in poultry in terms of publications, top contributing authors, funding agencies, institutional affiliations, and corresponding authorship. The network analysis also revealed that China significantly cooperated with other countries including United States, Egypt, Turkey, Germany, and Australia. In terms of co-authorship, citations, and bibliographic coupling, China had the greatest connections, networks, and TLS with other countries. This may be closely attributed to the significant financial investments by the Chinese government to support research on heat stress in poultry. The study revealed that out of the top 10 funding agencies, five were sponsors from China including the National Natural Science Foundation of China, National Key Research and Development Program of China, and Natural Science Foundation of Jiangsu Province. Evidently, the most productive institutions were also from China, with the most significant being the Nanjing Agricultural University. The institutional networks for co-authorship and citation analysis further revealed that Nanjing Agricultural University played a significant role in establishing strong research cooperation and collaboration with other institutions both within and outside China.

With respect to the authors, Kazim Sahin from Firat University, Turkey, Nurhan Sahin from Firat University, Turkey, and Feng Gao from Nanjing Agricultural University, China contributed the highest to global research on heat stress in poultry. Studies by Kazim Sahin and Nurhan Sahin were majorly centered on vitamin and mineral metabolism, quail production, phytochemicals, oxidative stress, and heat stress alleviation (Sahin et al., 2012; Sahin et al., 2013; Sahin et al., 2017; Orhan et al., 2021). Both authors were closely affiliated and collaborated on most of their research. On the other hand, Feng Gao focused on studies related to muscle metabolism, taurine supplementation, heat stress-induced oxidative damage, and the effects of chronic heat stress on broilers (Lu et al., 2017; Ma et al., 2018; Lu et al., 2019; Ma et al., 2021). Furthermore, the co-authorship analysis revealed that several of the authors, even those with a higher number of publications were excluded from the network. In line with this, the international co-authorship of authors was found to be 24.36%, thus suggesting that the inter-collaborative efforts of authors in this research field were relatively low. In addition, research works from Gao, Feng; Bao, Endong; Tang, Shu; Lamont, Susan J; and Alagawany Mahmoud were highly cited by other authors, while the works from Sahin, K., Yahav, S., Attia, Y.A., Quinteiro, W.M., and Lin, H. were among the top co-cited papers, suggesting that they contributed to the high impact papers sought after in the field. Consequently, documents from these authors were also found among the top globally cited research in this field (Sahin and Kucuk, 2003; Lin et al., 2006; Yahav, 2009; Attia et al., 2021). The bibliographic coupling of authors characterizes the phenomenon where two authors cite similar document (s) in the articles that they have published (Ma, 2012; Zhang and Yuan, 2022). Findings from both the citation and bibliographic coupling analysis showed that Bao, Endong and Tang, Shu who were affiliated with Nanjing Agricultural University, China had a strong connection in their bibliographic network, suggesting that these authors have close similarities in their research outputs. Altogether, key scholars who have contributed quantitatively or qualitatively to promoting research on heat stress in poultry were identified, indicating their substantial contributions to the field.

Bibliometric analysis of document sources (including journals, books, etc.) is important in providing updated insights and trends for a particular research area, as well as in guiding scholars to select suitable journals for their research communication (Cheng et al., 2022; Liu et al., 2022). Journals including Poultry Science, British Poultry Science, Journal of Thermal Biology, Animal, and Brazilian Journal of Poultry Science were the most prolific sources of articles on heat stress in poultry and they also accrued higher citation index. Journal co-citation analysis and bibliographic coupling further emphasized that Poultry Science had the largest network and was strongly connected with other journals such as Journal of Thermal Biology, World’s Poultry Science Journal, Animals, and British Poultry Science. These may be related to the publication of high-impact findings in these journals, thus suggesting that the hot topics and future trends emanating from research on heat stress in poultry are likely to be reported in these journals. Evidently, Poultry Science journal produced most of the top globally cited articles, revealing that this journal is a relevant, consistent, and authoritative source for high-caliber research and information related to poultry production.

The article, titled “Effect of heat stress on oxidative stress, lipid peroxidation and some stress parameters in broiler” by Altan O. et al. (2003) was identified as the most globally cited document on heat stress in poultry. Key findings of this study were that heat stress increased fearfulness, induced oxidative stress, and initiated significant physiological responses in broilers (Altan et al., 2003). Two review articles also contributed significantly to the global research progress on heat stress in poultry. They were “Strategies for preventing heat stress in poultry” by Lin H et al. (2006) and “Association between heat stress and oxidative stress in poultry; mitochondrial dysfunction and dietary interventions with phytochemicals” by Akbarian A. et al. (2016). Given the widespread concerns on how to mitigate the adverse impacts of heat stress in poultry production, Lin et al. (2006) was among the early reports to surmise various methods, which included, genetic, nutritional, feeding, and environmental strategies that can be adopted to cope with heat stress in poultry. In addition, following the discovery that oxidative stress was closely associated with heat stress in poultry, Akbarian et al. (2016) provided a detailed discussion that explained the mechanisms through which heat stress induces oxidative stress, and also presented the use of antioxidant phytochemicals as a nutritional strategy for the alleviation of mitochondrial dysfunction in heat-stressed birds. From the citation networks, documents including Altan et al. (2003), Bartlett (2003), Lin et al. (2006), Akbarian et al. (2016), and Sohail et al. (2010) had the greatest linkages and also contributed substantially to the advancement of research on heat stress in poultry. Several documents were commonly cited by scholars in this research area, especially the works of Lara and Rostagno (2013) which reported on the “Impact of heat stress on poultry production” and Quinteiro-Filho et al. (2010) which reported that “Heat stress impairs performance parameters, induces intestinal injury, and decreases macrophage activity in broiler chickens”.

Keywords are considered important terms and phrases from a research paper that rightly expresses the content covered, help generate new information and frontiers of knowledge, and assist in the retrieval of relevant content. Commonly, author-generated keywords (that is, Author keywords) are provided during article submission to describe the main contents/concepts addressed in their research. Also, the Keyword Plus is generated by Web of Science from the words and phrases that occur in the titles of cited references (Tripathi et al., 2018). Therefore the use of both author keywords and Keyword Plus explicitly expresses the knowledge structure, the article’s content, and the interactions of various research in a given subject area (Zhang et al., 2016). In this study, the top five keywords with the highest WordCloud frequency were “chickens”, “growth performance”, “supplementation”, “performance”, and “oxidative stress”. Word Cloud provides a visual representation of texts that are frequently occurring, such that the bigger and bolder a word appears, the higher its frequency of occurrence. Interestingly, the keyword “oxidative stress” had a higher occurrence than “heat stress” revealing a shift in research interest, which suggest that more researchers were gradually getting involved with studies that addressed oxidative stress in poultry. In addition, the thematic evolution of Keyword plus identified that research on oxidative stress had gained momentum over the years. Importantly, the thematic growth on oxidative stress had evolved to integrate other terms related to performance, supplementation, gene expression, and growth performance. This was also consistent with the overlay visualization which showed that heat stress co-occurred with various new topics such as “meat quality”, “antioxidants”, “microflora”, “intestinal barrier”, “expression”, and “rna-seq”.

The co-occurrence network of all keywords unveiled that current research on heat stress in poultry mainly focused on studies of growth performance, environment and production (red nodes), studies on intestinal morphology, and microbiota (green nodes), studies on the molecular mechanisms of heat stress (blue nodes), studies on immune response and metabolites, studies on meat quality and carcass characteristics (purple nodes), and studies on oxidative stress (aqua nodes). With the recent development in research, it is interesting to note that several terms have emerged in relation to heat stress such as “essential oils”, “resveratrol”, “betaine”, “curcumin”, “probiotic mix”, “flavonoids”, and “plant extracts”. These are important agents provided as supplements to poultry in a bid to alleviate the detrimental effects of heat stress. Several studies have demonstrated their protective and beneficial effects on the growth performance, behavior and welfare, immune system, antioxidant capacity, egg production, nutritional metabolism, intestinal health, thermotolerance, and other physiological variables of heat-stressed birds (Nawab et al., 2020; Sariozkan et al., 2020; Jiang et al., 2021; Liu et al., 2021; Mohammadi, 2021; Wen et al., 2021; Yang et al., 2021).

The thematic map analysis of keywords allows for the organization of keyword groupings according to their density and centrality into a single circle for mapping as a two-dimensional image (Ejaz et al., 2022). From the Keyword plus, it was identified that motor topics including “growth performance”, “supplementation”, “ascorbic acid research”, and “meat quality”, have been well-researched with respect to heat stress in poultry (Mahmoud et al., 2004; Abidin and Khatoon, 2013; Kumar et al., 2021; Piray and Foroutanifar, 2021). However, some topics that were weakly developed from both the emerging and declining themes (microflora, intestinal morphology, and probiotic mixture) and the basic themes (lipid-peroxidation, egg production, and ambient temperature) could be of research interest for further studies on heat stress in poultry. In examining the Author keywords, some underdeveloped and marginally important topics grouped into the emerging and declining themes were “hsp70”, “rna-seq”, “co-enzyme q10”, and “transcriptome”. To address this, studies have reported that co-enzyme q10 protects the chicken primary myocardial cells against damage and apoptosis during *in vivo* heat stress by upregulating hsp70 expression and HSF1 binding activity (Xu et al., 2017a; Xu et al., 2017b; Xu et al., 2019b), and that co-enzyme q10 induced autophagy and suppressed the PI3K/AKT/mTOR signaling as a protective mechanism against heat stress (Xu et al., 2019a). Alongside this, several studies have employed the whole transcriptomics approach, as well as other RNA sequencing technology to investigate the effects of heat stress in poultry (Saelao et al., 2018; Te Pas et al., 2019; Kim et al., 2021; Liang et al., 2021). With the advent of this tool, the molecular mechanisms that occur in distinct cells or tissues of various poultry species during heat stress exposure can be profiled, quantified, and comprehensively understood, thus presenting useful information to advance research in this field. The transcriptomics approach has been utilized to investigate the effects of heat stress on various tissues including the liver (Jastrebski et al., 2017; Wang et al., 2020), bursa (Monson et al., 2018; Chanthavixay et al., 2020), thymus (Monson et al., 2019), cardiac and skeletal muscle (Srikanth et al., 2019), hypothalamus (Sun et al., 2015), and heart (Zhang et al., 2017; Zhang et al., 2019). In addition, some topics which are relevant but weakly developed were identified in the basic theme, such as “corticosterone”, “acute heat stress”’ “chronic heat stress”, “oxidative stress”, “feed restriction, “meat quality”, and “gene expression”. Importantly, these topics may be considered highly relevant and of great potential in stimulating future research and scientific development on heat stress in poultry.

To the best of our knowledge, the present study is the first to examine the global research status and emerging trends on heat stress in poultry species using the bibliometric approach. Noteworthy, research progress in this field is gradually developing with greater growth potential. Presently, the need for extensive global cooperation between authors, institutions, and countries cannot be overemphasized. Current research on heat stress in poultry could be identified as focusing on production performance, physiological responses, oxidative stress, and dietary supplements. However, it is deduced that future research trends will focus more on topics such as; “antioxidants”, “meat quality”, “microflora”, “intestinal barrier”, “rna-seq”, “animal welfare”, “gene expression”, “probiotics”, “feed restriction”, and “inflammatory pathways”. Therefore, this comprehensive analysis of the current status and emerging topics on heat stress in poultry would serve as a useful reference for researchers, policymakers, and other stakeholders in the poultry industry.

## 5 Limitations of the study

It is important to point out that despite the novelty of this work and the important findings established from this study, there are a few limitations associated with it.

1. The documents retrieved for this study were solely based on the WoSCC database, as such not inclusive of other database collections such as Scopus, Pubmed, Dimension *etcetera*, which may have more information to deliver. However, it is agreed that Web of Science is the most suitable database and the most recommended for bibliometric studies in a specific field (Cheng et al., 2022; Shan et al., 2022).
2. The documents mined from the search may not necessarily obtain all the related literature due to the diversity of keywords. However, the detailed search strategy employed, the advanced search technique, and the logical use of Boolean operators have helped to improve the relevance and accuracy of retrieved results.
3. The key term in this study “heat stress in poultry” was expressed as “heat stress” AND “Poultry”, with the use of “AND” as a Boolean operator to ensure that all related keywords were retrieved and the search result was narrowed down to only the relevant terms.

## Data Availability

The original contributions presented in the study are included in the article/Supplementary Material, further inquiries can be directed to the corresponding authors.
