# Supplementary material for: Global trends and research frontiers on heat stress in poultry from 2000 to 2021: A bibliometric analysis
Source: Front Physiol. 2023 Feb 7;14:1123582. doi: 10.3389/fphys.2023.1123582 (PMC9941544; doi:10.3389/fphys.2023.1123582)
Supplement: Supplementary file 1 [file DataSheet1.docx]

Supplementary Material

Global Trends and Research Frontiers on Heat Stress in Poultry from 2000 to 2021: A Bibliometric Analysis

Victoria Anthony Uyanga^1*^, Taha H. Musa^2^, Emmanuel O. Oke^3^, Jingpeng Zhao^1^, Xiaojuan Wang^1^, Hongchao Jiao^1^, Okanlawon M. Onagbesan^3^ and Hai Lin^1*^

^1^College of Animal Science and Technology, Shandong Provincial Key Laboratory of Animal Biotechnology and Disease Control and Prevention, Key Laboratory of Efficient Utilization of Non-grain Feed Resources (Co-construction by Ministry and Province), Ministry of Agriculture and Rural Affairs, Shandong Agricultural University, Tai’an 271018, China.

^2^ Biomedical Research Institute, Darfur University College, Nyala, South Darfur, Sudan

^3^Department of Animal Physiology, Federal University of Agriculture, Abeokuta P.M.B. 2240, Ogun State, Nigeria

*** Correspondence:**Hai Lin: hailin@sdau.edu.cn; Tel: 86 538 8249203; Fax: 86 538 8241419

Victoria Anthony Uyanga: uyangava@sdau.edu.cn; Tel: 86 150 6985 7298

# Supplementary Data

**SEARCH STRATEGY ON WOSCC FOR HEAT STRESS AND POULTRY -10.10.2022**

1. Results for "poultry" (Title) OR "chicken" (Title) OR "guinea fowl" (Title) OR "turkey" (Title) OR "geese" (Title) OR "duck" (Title) OR "quail" (Title) OR "pigeon" (Title) OR "broilers" (Title) OR "laying hen" (Title) OR "fowl" (Title) OR "ostriches" (Title) OR "pheasants" (Title)

**182,285 results from Web of Science Core Collection**

1. Results for "heat stress" (Title) OR "thermal stress" (Title) OR "high temperature" (Title) OR "hot temperature" (Title) OR "thermal condition" (Title) OR "high ambient temperature" (Title) OR "heat exposure" (Title) OR "heat conditioning" (Title) OR "high environmental temperature" (Title)

# 111,283 results from Web of Science Core Collection

1. Advanced Search= Results for #1 AND #2

# 684 results from Web of Science Core Collection for:


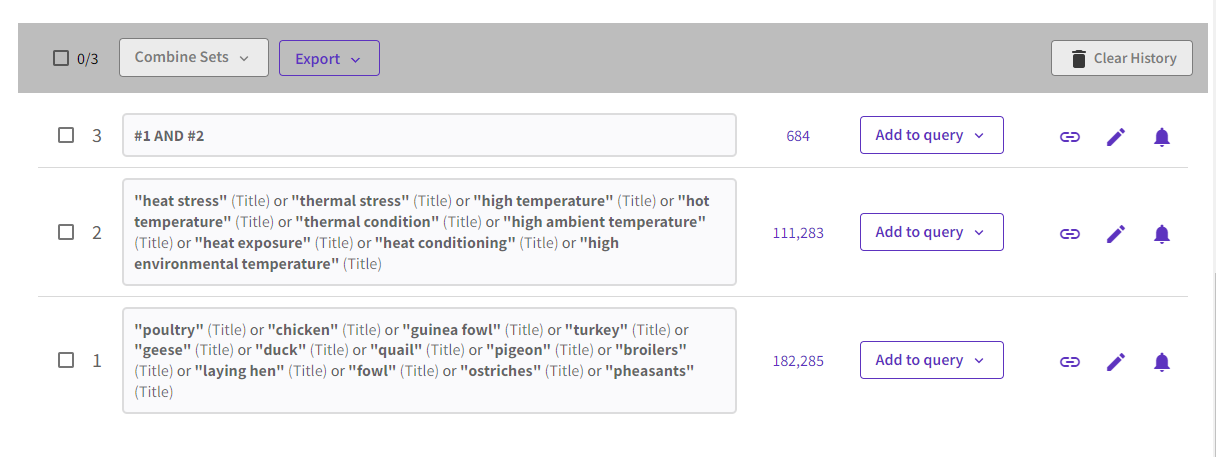


1. Results for #1 AND #2 and 2000 or 2001 or 2002 or 2003 or 2021 or 2020 or 2019 or 2018 or 2017 or 2016 or 2015 or 2014 or 2013 or 2012 or 2011 or 2010 or 2009 or 2008 or 2007 or 2006 or 2005 or 2004 (Publication Years)

# 520 results from Web of Science Core Collection for:

1. Refine by document type = Article or review article

# 486 results from Web of Science Core Collection for:


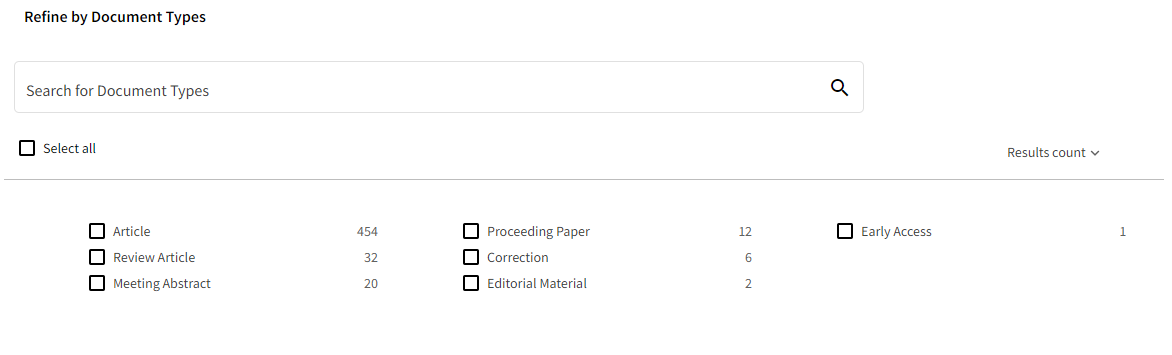


1. **Refine by Languages**


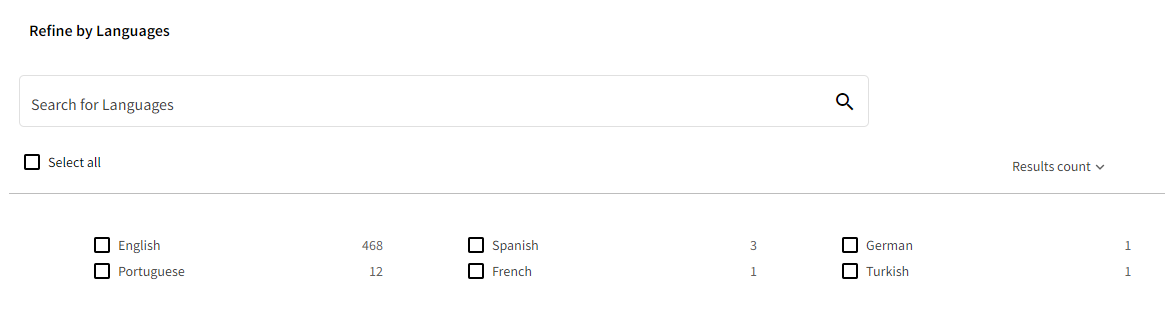


# 468 results from Web of Science Core Collection for:

Analyze Results: #1 AND #2 and 2000 or 2001 or 2002 or 2003 or 2021 or 2020 or 2019 or 2018 or 2017 or 2016 or 2015 or 2014 or 2013 or 2012 or 2011 or 2010 or 2009 or 2008 or 2007 or 2006 or 2005 or 2004 (Publication Years) and Article or Review Article (Document Types) and English (Languages)

1. Web of science index : All WOS index were used for file selection = 468 total


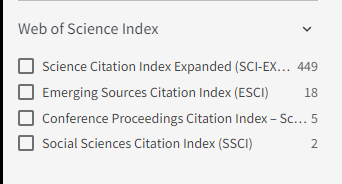


1. Exported file types


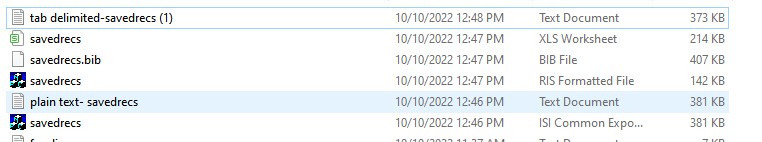


# Supplementary Table 1

**Thematic evolution of Keyword Plus of articles on heat stress and Poultry from 2000 to 2021**

| **Cluster** | **Callon Centrality** | **Callon Density** | **Rank Centrality** | **Rank Density** | **Cluster Frequency** |
| --- | --- | --- | --- | --- | --- |
| ***Time series 1 (2000-2005)*** |  |  |  |  |  |
| Chickens | 3.320238095 | 71.62698413 | 8 | 5 | 46 |
| Temperature | 1.016904762 | 111.25 | 3 | 8 | 24 |
| Egg-production | 1.372222222 | 96.80555556 | 6 | 7 | 31 |
| Corticosterone | 1.023333333 | 45 | 4 | 1 | 7 |
| Laying hens | 0.733333333 | 62.5 | 2 | 3 | 13 |
| Chicks | 1.527777778 | 90 | 7 | 6 | 11 |
| Environmental-temperature | 1.111111111 | 63.88888889 | 5 | 4 | 7 |
| Oxidative stress | 0 | 50 | 1 | 2 | 2 |
| ***Time series 2 (2006-2010)*** |  |  |  |  |  |
| Chickens | 4.750793651 | 83.94097222 | 8 | 7 | 53 |
| Performance | 1.403174603 | 56.71296296 | 3 | 4 | 16 |
| Lipid-peroxidation | 0.969047619 | 89.28571429 | 1 | 8 | 13 |
| Environmental-temperature | 1.844166667 | 79.64285714 | 6 | 6 | 19 |
| High ambient-temperature | 1.672222222 | 56 | 5 | 3 | 16 |
| Domestic-fowl | 2.402777778 | 59.02777778 | 7 | 5 | 16 |
| Oxidative stress | 1.291666667 | 43.75 | 2 | 2 | 6 |
| Stress | 1.555555556 | 38.88888889 | 4 | 1 | 6 |
| ***Time series 3(2011-2015)*** |  |  |  |  |  |
| Chickens | 7.387932365 | 71.08260007 | 6 | 4 | 112 |
| Growth-performance | 3.745780423 | 76.62358277 | 4 | 5 | 54 |
| Supplementation | 8.266869949 | 82.82471541 | 7 | 6 | 150 |
| Performance | 6.673931188 | 62.89754363 | 5 | 2 | 66 |
| Gene-expression | 2.64702381 | 106.420068 | 3 | 7 | 36 |
| Chicken skeletal-muscle | 1.166666667 | 69.21296296 | 2 | 3 | 16 |
| Age | 0.25 | 62.5 | 1 | 1 | 4 |
| ***Time series 4 (2016-2021)*** |  |  |  |  |  |
| Growth-performance | 9.532096704 | 35.38779358 | 6 | 2 | 518 |
| Chickens | 7.204470812 | 38.08690696 | 5 | 5 | 395 |
| Oxidative stress | 6.189537752 | 39.16332728 | 3 | 6 | 393 |
| Broiler-chickens | 6.857814749 | 35.61917387 | 4 | 3 | 260 |
| Temperature | 2.619852261 | 33.15942303 | 2 | 1 | 104 |
| Carcass | 1.800601852 | 37.84018759 | 1 | 4 | 55 |
